# *Bartonella* Species and Trombiculid Mites of Rats from the Mekong Delta of Vietnam

**DOI:** 10.1089/vbz.2014.1604

**Published:** 2015-01-01

**Authors:** Hoang Kim Loan, Nguyen Van Cuong, Ratree Takhampunya, Kewalin Klangthong, Lynn Osikowicz, Bach Tuan Kiet, James Campbell, Juliet Bryant, Sommai Promstaporn, Michael Kosoy, Nguyen Van Hoang, Serge Morand, Yannick Chaval, Vo Be Hien, Juan Carrique-Mas

**Affiliations:** ^1^ Institute Pasteur, Ho Chi Minh City, Vietnam.; ^2^ Oxford University Clinical Research Unit, Vietnam.; ^3^ AFRIMS, Bangkok, Thailand.; ^4^ Centre for Disease Control, Fort Collins, Colorado.; ^5^ Sub-Department of Animal Health, Dong Thap, Vietnam.; ^6^ Institute Nationale de la Recherche Agricole, Montpellier, France.

**Keywords:** *Bartonella*, *Rattus*, Chiggers, Vietnam

## Abstract

A survey of *Bartonella* spp. from 275 rats purchased in food markets (*n*=150) and trapped in different ecosystems (rice field, forest, and animal farms) (*n*=125) was carried out during October, 2012–March, 2013, in the Mekong Delta of Vietnam. The overall *Bartonella* spp. prevalence detected by culture and PCR in blood was 14.9% (10.7–19.1%), the highest corresponding to *Rattus tanezumi* (49.2%), followed by *Rattus norvegicus* (20.7%). Trapped rats were also investigated for the presence and type of chiggers (larvae of trombiculid mites), and *Bartonella* spp. were investigated on chigger pools collected from each rat by RT-PCR. A total of five *Bartonella* spp. were identified in rats, three of which (*B. elizabethae*, *B. rattimassiliensis*, and *B. tribocorum*) are known zoonotic pathogens. Among trapped rats, factors independently associated with increased prevalence of *Bartonella* spp. included: (1) Rat species (*R. tanezumi*); (2) the number of Trombiculini–Blankaartia and Schoengastiini–Ascoschoengastia mites found on rats; and (3) the habitat of the rat (*i.e*., forest/fields vs. animal farms). The prevalence of *Bartonella* infection among chiggers from *Bartonella* spp.–positive *R. tanezumi* rats was 5/25 (25%), compared with 1/27 (3.7%) among *Bartonella* spp.–negative *R. tanezumi* rats (relative risk [RR]=5.4, 95% confidence interval [CI] 0.68–43.09). The finding of *Bartonella* spp.–positive chiggers on *Bartonella* spp.–negative rats is strongly suggestive of a transovarial transmission cycle. Rats are ubiquitous in areas of human activity and farms in the Mekong Delta; in addition, trapping and trading of rats for food is common. To correctly assess the human risks due to rat trapping, marketing, and carcass dressing, further studies are needed to establish the routes of transmission and cycle of infection. The widespread presence of these zoonotic pathogens in rats and the abundance of human—rat interactions suggest that surveillance efforts should be enhanced to detect any human cases of *Bartonella* infection that may arise.

## Introduction

The genus *Bartonella* contains intracellular Gram-negative bacteria considered to be opportunistic human pathogens, although some species are capable of causing disease in healthy people. Infections with *Bartonella* spp. are associated with a remarkable variety of clinical symptoms, ranging from transitory febrile disease, to severe vascular dysfunction and endocarditis (Maguiña and Ordaya 2010). Although there is a degree of host specificity, *Bartonella* spp. have a wide range of mammalian animal hosts, including felines, canines, primates, rodents, bats, and shrews (Breitschwerdt and Kordick 2000, Chomel and Kasten 2010). Depending on the *Bartonella* and host species, transmission between vertebrate hosts, including humans, occurs through arthropod vectors such as ticks, fleas, sand flies, and lice (Boulouis et al. 2005, Chomel and Kasten 2010, Kamani et al. 2013). Cases of infection with *Bartonella* spp. in humans have increasingly been reported from East and Southeast Asian countries (Kosoy et al. 2008, Paitoonpong et al. 2008, Bhengsri et al. 2010, Kosoy et al. 2010, Pachirat et al. 2011, Bai et al. 2012, Lim et al. 2012). Recent studies carried out in a number of Southeast Asian countries have detected a range of *Bartonella* spp. in rats (Castle et al. 2004, Bai et al. 2009, Bhengsri et al. 2010, Kosoy 2010, Loan et al. 2011). In Vietnam, a 2011 study on rats indicated an overall prevalence for *Bartonella* spp. of 19.2%, including zoonotic species such as *B. elizabethae,* and *B. rattimassiliensis* (Loan et al. 2011), both of which have been associated with febrile disease in southeast Asia (Kosoy et al. 2010). Data from Thailand suggest that exposure to ticks, outdoor activities, and exposure to rats are factors associated with human infection with *Bartonella* spp. (Bhengsri et al. 2010).

Chiggers are larval mites of the family Trombiculidae and are thought to be the most common ectoparasites on rats in the Mekong Delta, in contrast with ticks, lice, and fleas, which are rarely seen (unpublished information). The life cycle of trombiculid mites consists of three active stages—larvae (chiggers), nymphs, and adults. The nymph and adult stages are free-living, whereas the larvae are parasitic on a wide range of animals, including rats, squirrels, shrews, and birds (Traub and Wisseman 1974). In rats, chiggers are usually found attached to the tragus of the ear. About 350 species of chiggers have been described in Southeast Asia (Nadchatram and Dohany 1974). Chiggers of the genus *Leptotrombidium* are vectors of *Orientia tsutsugamushi*, the causative agent of scrub typhus (Traub and Wisseman 1974, Lerdthusnee et al. 2002). In Thailand, chiggers of the genera *Leptotrombidium*, *Schoengastia*, and *Blankarrtia* collected from rats were found to harbor zoonotic *Bartonella tamiae* (Kabeya et al. 2010).

The prevalence of *Bartonella* spp. in different rat species and their potential association with their ectoparasites have not been investigated in the Mekong Delta region of Vietnam, a region where rats are a commonly captured after rice harvests and sold for food (Phan et al. 2005). We conducted a survey of rats, including both specimens sold in live in markets as well as rats captured across the Mekong Delta with the aims to: (1) Investigate the prevalence and species diversity of *Bartonella* spp. in different species of rats; (2) characterize the burden and species of chiggers in rats and the prevalence of *Bartonella* spp. in the chiggers themselves; and (3) investigate variables associated with prevalence of *Bartonella* spp. (rat species, stage of development, as well as chigger burden and species).

## Materials and Methods

### Rat collection and processing

Rats were purchased from five provincial and district markets, in addition to four local traders (*i.e*., nine sources) located in five provinces of the Mekong Delta (Dong Thap, Tien Giang, An Giang, Vinh Long, and Can Tho) (∼30 per province) during October of 2012. Rat trapping was carried out in different locations in the province of Dong Thap during March, 2013. A total of 30 lines (one per location) of 10 live cage metal tomahawk traps were set up, checked for rats trapped, and reset over 10 subsequent mornings (totaling about 3000 trap-days). Traps were placed in farm sites where poultry and pigs were kept (*n*=20), as well as in crop fields (rice) and forest locations (*n*=10). Traps with a rat inside were brought back to the laboratory and were replaced by a new trap. On the day of purchase/catching, the following procedures were carried out on all rats: (1) Humane culling by overdose of inhalant anesthetic (isoflurane) following American Veterinary Medical Association (AVMA) guidelines (American Veterinary Medical Association 2007); (2) measurement of main biological and morphometric parameters (weight, sex, body length, tail length, foot length, ear length, skull length, ear length, fur color, reproductive status) to allow species identification; (3) postmortem aseptic collection of 1–3 mL of whole blood by cardiac puncture; and (4) collection of one ear from each animal, by cutting it from the base and placing into a 5-mL universal tube with 70°C ethanol. Detailed methods for sample collection are described elsewhere (Ceropath 2011).

### Rat species identification

The rat species identification was carried out on the basis of morphological characteristics. One rat representative of each batch (markets) and two to three of each species identified were confirmed by amplification of a conserved housekeeping gene (*cytochrome oxidase subunit I, COI*) using PCR followed by molecular sequencing of the product and blasting using the RatSEA barcoding tool (www.ceropath.org/barcoding_tool/ratsea).

### Identification of trombiculid mites

Rat ears were placed on separate Petri dishes containing 70% ethanol, and chiggers were counted under a stereomicroscope (magnification, 20×) after being removed using a fine needle. Chiggers were washed several times with distilled water to remove alcohol. Chiggers were then submerged in a drop of mounting fluid (Berlese's fluid-gum chloral, TCS Biosciences Ltd, UK) that had previously been placed on a glass slide. Slides were dried overnight using an incubator at 40°C and were then sealed with nail polish. Chiggers were examined using a phase-contrast microscope at 400×magnification. At least five chiggers from each ear were identified morphologically. The chigger mites were identified down to the subfamily–tribe–genus–subgenus based on published guidelines on Southeast Asian chiggers (Acari, Prostigmata, Trombiculidae) (Nadchatram and Dohany 1974).

### Culture of *Bartonella* spp. from rats

Blood was cultured for *Bartonella* spp. in accordance with the Centers for Disease Control and Prevention (CDC) procedures (Ying et al. 2002, Bai et al. 2009). Briefly, whole blood was diluted 1:4 in brain heart infusion media containing 5% amphotericin B (Sigma) to reduce the likelihood of fungal contaminants because of the slow growth of *Bartonella* spp. Diluted blood samples were pipetted onto Columbia agar (BioRad) plates containing 5% fresh sheep blood previously screened to ensure absence of *Bartonella* spp. Plates were incubated at 35°C in an atmosphere of 5% CO_2_ for up to 4 weeks. Plates were examined once per week for bacterial growth. Bacterial colonies were selected on the basis of colony morphology, size and shape, and Gram-staining characteristics, and were subcultured from initial plates and subsequent passages until a pure culture was obtained.

### Confirmation of *Bartonella* spp. by PCR

DNA extractions were performed on whole bacterial cells using a DNA extraction kit (QIAamp^®^ DNA Mini Kit, cat. no. 51304, Qiagen, The Netherlands). Primers BhCS871.p and BhCS1137.n for amplification product of 379 of the citrate synthase (*gltA*) gene of *Bartonella* were used. The PCR was carried out in a Techne 512 Thermal Cycler (Techne, UK) following program parameters—an initial denaturing at 95°C for 5 min, and 35 cycles at 95°C for 1 min, 56°C for 1 min, and 72°C for 1 min. The PCR products were analyzed for the presence of amplicons of the correct size by electrophoresis in 1.5% agarose gels containing ethidium bromide.

### Detection of *Bartonella* spp. in chiggers

Chiggers from each rat were pooled and subjected to genomic DNA extraction using a modified tissue protocol from QIAamp^®^ DNA Mini Kit (Qiagen, Hilden, Germany). Chiggers were placed in a 1.5-mL microcentrifuge tube and punctured with a fine needle. Ninety microliters of ATL tissue lysis buffer were added, and the sample was either processed immediately or stored at −70°C. Ten microliters of Proteinase K solution (20 mg/mL) were added, and the sample was incubated at 56°C for 3 h. Subsequently, 100 μL of AL buffer was added, and the sample was mixed by pulse-vortexing for 15 s, followed by incubation at 70°C for 10 min. Then 100 μL of absolute ethanol was added, and the sample was mixed by pulse-vortexing for 15 s. The sample was applied to a QIAamp spin column, and DNA was eluted in 50 μL of AE buffer and stored at −20°C until amplification. Detection of *Bartonella* in chiggers was performed by RT-PCR following a previously published protocol using *Bartonella* specific primers targeting the transfer-mRNA gene (*ssrA*) (Diaz et al. 2012).

### *Bartonella* species identification

Sequences were analyzed using Lasergene v. 8 sequence analysis software (DNASTAR Inc., Madison, WI) to determine consensus of sequences for the amplified region of the *gltA* gene. Unique sequences obtained in this study were submitted to GenBank. The Clustal V program within the MegAlign module of Lasergene was used to compare homologous *Bartonella* spp. *gltA* sequences from the present study and from GenBank. A criterion of ≥96% homology to *gltA* was used to define groups (Gundi et al. 2009).

### Statistical analyses

*Bartonella* spp. prevalence comparisons were performed using chi-squared tests. For trapped rats, the association between *Bartonella* spp. and the species, body length, type of habitat (farm vs. field/forest), and number of chiggers (log-transformed) was explored by multivariable logistic regression modeling. Body length was transformed into a binary variable (large/small) based on a cutoff determined by the 50% quantile for each species. Multivariable model building was carried out in a stepwise forward fashion starting with variables with the lowest *p* value in univariable analyses (Hosmer et al. 2004). Interaction terms between all main effects remaining in the model were investigated. All data analyses were carried out using R software (www.r-project.org).

## Results

### Prevalence of *Bartonella* spp. in rats

A total of 275 rats were examined, of which 150 were purchased in markets and 125 were trapped. Five different rat species were identified: *Rattus argentiventer* (*n*=104), *Bandicota indica* (*n*=65), *R. tanezumi* (*n*=61), *R. norvegicus* (*n*=29), and *R. exulans* (*n*=16). All rat species were present in both collections, except for *R. exulans*, which was not represented in the market collection. *R. argentiventer* was more represented in the market collection (58.7%) than among trapped rats (12.8%). In contrast, *R. tanezumi* was the most commonly trapped rat species (41.6%), but represented only 6% of market rats. A total of 41 *Bartonella* strains were identified by culture and further confirmed by PCR. We did not observe any evidence of colonies of different morphology indicative for co-infection by different *Bartonella* species in the same rat.

The overall *Bartonella* spp. prevalence in rats was 14.9% (95% confidence interval [CI]=10.7–19.1%). The prevalence in rats purchased in markets was 8.7% compared with a prevalence of 22.4% among trapped rats (χ^2^=9.08; *p*=0.002). The highest prevalence corresponded to *R. tanezumi* (49.2%), followed by *R. norvegicus* (20.7%). *B. indica* and *R. argentiventer* rats had <5% *Bartonella* spp. prevalence, and all 16 *R. exulans* rats were *Bartonella* spp.–negative. For *R. tanezumi*, the *Bartonella* spp. prevalence was highest among rats trapped in fields/forests habitats (63.6%), and lowest among rats trapped on farms (21.0%) (Table 1).

**Table 1.** Prevalence of *Bartonella* spp. among Rats Purchased from Markets and Captured in Different Habitats of the Mekong Delta, October, 2012–March, 2013)

		*Rat species*					
*Site*		B. indica	R. tanezumi	R. argentiventer	R. exulans	R. norvegicus	*Total*
Market	Pos./Total	1/44	5/9	3/88	—	4/9	13/150
	% Pos. (95% CI)	2.3 (0.0–6.7)	55.5 (23.1–88.0)	3.4 (0.0–7.2)	—	44.4 (12.0–76.9)	8.7 (4.2–13.2)
Farms	Pos./Total	0/10	4/19	0/14	0/15	2/18	6/76
	% Pos. (95% CI)	0.0 (N.C.)	21.0 (0–61.0)	0.0 (N.C.)	0.0 (N.C.)	11.1 (0–54.7)	7.9 (1.8–14.0)
Field/forest	Pos./Total	1/11	21/33	0/2	0/1	0/2	22/49
	% Pos. (95% CI)	9.1 (0–65.4)	63.6 (43.1–84.2)	0.0 (N.C.)	0.0 (N.C.)	0.0 (N.C.)	44.9 (30.9–58.8)
All rats	Pos./Total	2/65	30/61	3/104	0/16	6/29	41/275
	% Pos. (95% CI)	3.1 (0.0–7.3)	49.2 (36.6–61.7)	2.9 (0.0–6.1)	0.0 (N.C.)	20.7 (5.9–35.4)	14.9 (10.7–19.1)

Pos., positive; CI, confidence interval; N.C., not calculated.

### Distribution of *Bartonella* spp. in rats

**FIG. 1.**
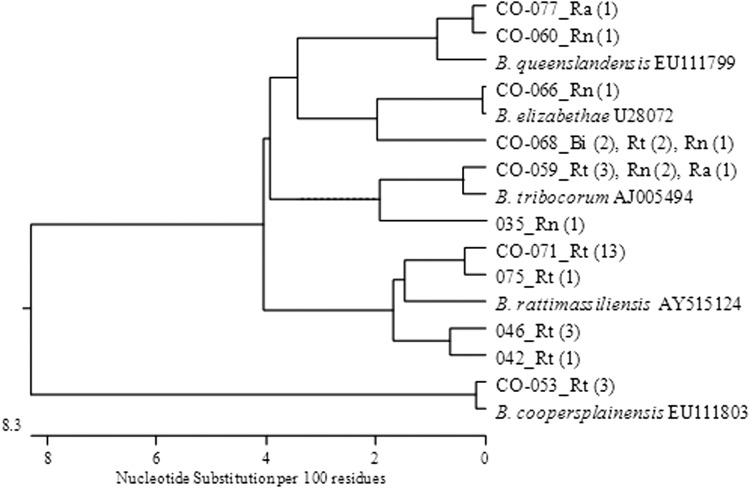
Phylogenetic tree showing relationship between sequences of 32 *Bartonella* spp. analyzed for the amplified region of the gltA (Mekong Delta, Vietnam, October, 2012–March, 2013).

The species of *Bartonella* spp. was successfully determined for 32/41 *Bartonella* isolates (Fig. 1). In four cases, there was insufficient DNA available for sequencing, and, in five cases, the sequencing results resulted in nonspecific reactions. A total of five different species were identified. The most commonly identified species were *Bartonella rattimassiliensis*, *B. tribocorum*, and *B. elisabethae* (corresponding to 43.8%, 21.9%, and 18.8% of rats infected with *Bartonella* spp.). *B. rattimassiliensis* and *B. coopersplainensis* were found only in *R. tanezumi*, whereas all other *Bartonella* spp. were found at least in two different host species (Table 2).

**Table 2.** Distribution of *Bartonella* spp. Identified in Rats (Mekong Delta, Vietnam, October, 2012–March, 2013)

	*Rat species*					
Bartonella *spp.*	R. tanezumi	R. norvegicus	B. indica	R. argentiventer	*All rats*	*Percent (%)*
*B. rattimassiliensis*	14	0	0	0	14	43.8
*B. tribocorum*	3	3	0	1	7	21.9
*B. elizabethae*	2	2	2	0	6	18.8
*B. coopersplainensis*	3	0	0	0	3	9.4
*B. queenslandensis*	0	1	0	1	2	6.3
Total isolates	22	6	2	2	32	100.0

### Trombiculid larvae (chiggers) in trapped rats

**FIG. 2.**
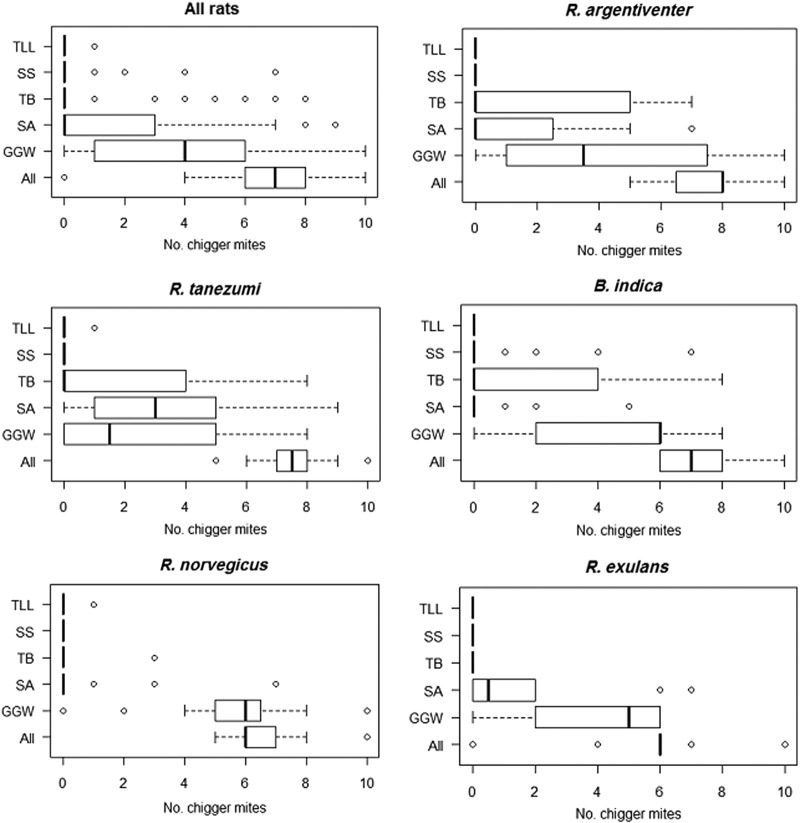
Number of chigger mites per animal by rat species (counts on one ear). (Mekong Delta, Vietnam, October, 2012–March, 2013). Tribe–genus–subgenus): TTL, Trombiculini–Leptotrombidium–Leptotrombidium; SS, Schoengastiini–Schoengastia; TB, Trombiculini–Blankaartia; SA, Schoengastiini–Ascoschoengastia; GGW, Gahrliepiini–Gahrliepia–Walchia.

All rats investigated except one (*R. exulans*) contained at least one chigger in their ears. The main trombiculid species identified corresponded to (tribe–genus–subgenus): Gahrliepiini–Gahrliepia–Walchia (GGW), Schoengastiini–Ascoschoengastia (SA), Trombiculini–Blankaartia (TB), Schoengastiini–Schoengastia (SS), and Trombiculini–Leptotrombidium–Leptotrombidium (TLL). Overall counts were similar for all rat species (median six to eight). *R. argentiventer* rats had the highest chigger counts (median 8; interquartile range [IQR]=6.7–8.0), followed by *R. tanezumi* (median 7; IQR=7–8), whereas *R. exulans* had the lowest (median 6; IQR=6.0–6.0) (Fig. 2).

### Prevalence of *Bartonella* spp. in chiggers

Thirteen chigger pools of 124 (10.5%) tested positive for *Bartonella* spp. A total of 6/28 (21.4%) chigger pools from *Bartonella* spp.–positive rats tested positive, compared with 7/96 (7.3%) pools from *Bartonella* spp.–negative rats (relative risk [RR]=2.97, 95% CI=1.09–8.12). The prevalence of *Bartonella* spp. in chiggers from *Bartonella* spp.–positive *R. tanezumi* rats was 5/25 (25%), compared with 1/27 (3.7%) among *Bartonella* spp.–negative *R. tanezumi* rats (3.7%) (RR=5.4, 95% CI=0.68–43.09).

### Risk factors for *Bartonella* spp. positivity in rats

Factors independently associated with *Bartonella* spp. prevalence in rats were: (1) Rat species (*R. tanezumi* vs. other) (odds ratio [OR]=7.68); (2) presence of SA and TB chiggers (log-transformed) (OR=2.23 and OR=2.18, respectively); and (3) type of habitat (rat trapped in field/ forest vs. trapped in a farm) (OR=3.20). Large rats (relative to the median for each species) had a higher risk of positivity (OR=2.55), although it was not significant (*p*=0.103) and was therefore excluded from the model. Interaction terms between main effects were not significant (*p*>0.05) (Table 3).

**Table 3.** Results from Multilevel Logistic Regression Model with Significant Factors for *Bartonella* spp. Prevalence among Rats Trapped in Dong Thap, February–March, 2013

	*OR*	*95% CI*	p *value*
*Rattus tanezumi* (baseline=other species)	7.68	1.78–33.16	0.006
Log(SA+1)	2.23	0.98–5.11	0.051
Log(TB)+1	2.18	1.02–4.69	0.043
Rat trapped in forest/field (baseline=farm)	3.20	0.93–10.94	0.064

Model intercept: −4.31 (standard error, 0.76).

OR, odds ratio; CI, confidence interval; SA, Schoengastiini–Ascoschoengastia; TB, Trombiculini–Blankaartia.

### Size of *R. tanezumi* and *Bartonella* spp. prevalence

**FIG. 3.**
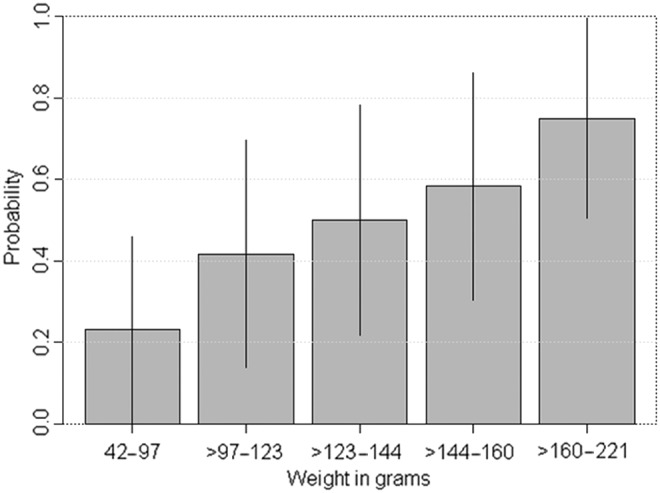
*Bartonella* spp. prevalence by body length among *R. tanezumi* rats (*n*=61) (Mekong Delta, Vietnam, October, 2012–March, 2013).

Among *R. tanezumi*, there was a statistically significant association between body length and the probability of testing positive for *Bartonella* spp. (χ^2^ for trend=8.54, *p*=0.003) (Fig. 3). In contrast, among *R. norvegicus*, the body length among *Bartonella* spp.–positive rats was smaller (189.0 mm) than for *Bartonella* spp.–negative individuals (168.5 mm) (*t*=27.42; *p*<0.01).

## Discussion

The observed overall *Bartonella* spp. prevalence in rats (14.9%) was comparable with a previous study in Vietnam (19.2%) (Loan et al. 2011) and with studies from other East and Southeast Asian countries (range 7.1–50%) (Castle et al. 2004, Winoto et al. 2005, Lin et al. 2008, Angelakis et al. 2009, Bai et al. 2009, Hsieh et al. 2010, Loan et al. 2011, Jiyipong et al. 2012). Our study identified differences in *Bartonella* spp. prevalence by rat species, with the observed highest prevalence (∼50%) corresponding to *R. tanezumi*. Studies in the Southeast Asian region and China have confirmed high levels of *Bartonella* spp. infection among *R. tanezumi* (Ying et al. 2002, Winoto et al. 2005, Loan et al. 2011).

All five *Bartonella* species identified in our study (*B. elizabethae*, *B. rattimassiliensis*, *B. tribocorum*, *B. queenlandensis*, and *B. coopersplainensis*) have previously been identified in rats from the region (Castle et al. 2004, Angelakis et al. 2009, Bai et al. 2009, Loan et al. 2011, Jiyipong et al. 2012). Four species (*B. elizabethae*, *B. rattimassiliensis*, *B. tribocorum*, and *B. queenlandensis*) are phylogenetically close and belong to the *B. elizabethae* complex sensu lato as an assemblage of genospecies and strains associated with Old World rats (Kosoy et al. 2012). Although phylogenetically more distant, *B. coopersplainensis* is also specific for Old World rats, but has been recently detected in an endemic Australian rat (*Rattus leucopus*) (Gundi et al. 2009). *B. rattimassiliensis* and *B. coopersplainensis* were found only in *R. tanezumi,* whereas *B. tribocorum*, *B. queenslandensis*, and *B. elizabethae* were harbored by rats of different species. *B. rattimassiliensis*, the most common *Bartonella* spp. identified in our study, has been isolated from *R. norvegicus* in Europe (Gundi et al. 2004) and from *R. tanezumi* trapped in other Mekong river countries (Jiyipong et al. 2012).

Three of the *Bartonella* spp. described (*B. elizabethae*, *B. rattimassiliensis*, and *B. tribocorum*) have been associated with human febrile illness in Thailand and have been linked epidemiologically to exposure to rats (Kosoy et al. 2010). Importantly, one of the *B. elizabethae* strains isolated from a *R. norvegicus* had an identical *gltA* gene to the strain isolated from a human endocarditis patient in Massachusetts (Daly et al. 1993). Other *Bartonella* species previously detected in human cases in Thailand, such as *B. henselae*, *B. tamiae,* and *B. vinsonii* subsp. *arupensis* (Paitoonpong et al. 2008, Kabeya et al. 2010, Bai et al. 2012), were not found in any of the rats examined in our study.

Our results suggest an increased prevalence of *Bartonella* spp. among older *R. tanezumi*. This suggests a cumulative risk and long-term carriage after infection, although the duration of persistence of infection in rats is unknown. These results contrast with previous studies of *Bartonella* spp. in other rat species, indicating that prevalence is inversely correlated with age (Fichet-Calvet et al. 2000, Kosoy et al. 2004, Jardine et al. 2006, Kosoy et al. 2008), although in a study on trapped *R. norvegicus* the prevalence of *B. rattimassiliensis* and *B. phoceensis* increased with rat weight (Gundi et al. 2004).

We observed a statistical association between *Bartonella* spp. positivity and the presence of Trombiculini–Blankaartia and Schoengastiini–Ascoschoengastia chiggers, suggesting that these species may play a role in the transmission of *Bartonella* spp. The finding of a higher prevalence of *Bartonella* spp. in rats with higher chigger burdens gives further support to this hypothesis, as trombiculid mites appear to be some of the most common ectoparasites on rats in the Mekong Delta. Unfortunately we could not confirm the identity of the *Bartonella* species in chiggers, because different genes were targeted on *Bartonella* spp. rats and chiggers (*gltA* and *ssrA*, respectively). The reason was a low sensitivity of detection of *Bartonella* spp. in chiggers based on *gltA* gene testing (data not shown).

These results should be interpreted with caution and should be followed by relevant studies to investigate the mites' vector competence and to establish the cycle of transmission (transovarial, transtadial, etc.). Trombiculid chiggers are thought to feed after attaching themselves firmly to their hosts' skin by a feeding tube (stylostome). Therefore, transmission of *Bartonella* spp. to the rat host would only be theoretically possible if transovarial transmission occurs. The finding of *Bartonella* spp.–positive chiggers on noninfected rats is strongly suggestive of transovarial transmission.

Because a number of trombiculid species have been reported to feed on humans (Shatrov and Kudryashova 2006), it is at least theoretically possible that infected chiggers that have not yet completed their attachment to the rodents' skin may migrate to a human during the process of catching, slaughtering, and dressing live rats. Therefore, these occupationally exposed communities may potentially be at risk should they not take the necessary precautions to protect themselves from chigger mite bites. Newly hatched chiggers questing for available hosts in the surroundings of rat nesting areas may also pose a risk to humans. *R. tanezumi*, and to a lesser extent *R. norvegicus*, which are common in areas of human dwelling may therefore represent the greatest risk.

To date no *Bartonella* spp. infections have been detected in patients in Vietnam. This is in part likely to be the result of limited awareness of this infection among the medical community, partly as a result of a diffuse, nonspecific clinical picture. We suggest that *Bartonella* spp. infections may be more common than previously thought in Vietnam given the widespread presence of rats in close proximity to human habitats, of which a fraction are infected with pathogenic *Bartonella* spp., as well as rat trade–related practices that may facilitate human contact with vectors of *Bartonella* spp. infection.
